# Activities of NEW Brain Aging

**DOI:** 10.1093/geroni/igab046.781

**Published:** 2021-12-17

**Authors:** Yeates Conwell

**Affiliations:** University of Rochester Medical Center, Rochester, New York, United States

## Abstract

The Network for Emotional Well-being and Brain Aging (NEW Brain Aging) was funded by NIA with the goal of forming a national, transdisciplinary collaborative that includes investigators with research expertise in emotional well-being (EWB), Alzheimer’s disease and related dementias (ADRD), human and animal neuroimaging, stress regulation, and computational/quantitative methods. Our objective is to stimulate mechanistic research identifying and testing mechanisms by which brain aging influences EWB and how EWB may impact risk for and progression of ADRD. This presentation will explain the structure and functions of the network that serve as a resource for investigators interested in EWB and aging research, and how to access them: a transdisciplinary community of scholars interested in brain, aging, and EWB research from both human and animal fields; webinars; workgroups to establish priorities for NEW Brain Aging activities; a resource repository; and pilot project funding opportunities to which network members can apply.

